# Urban Environment, Green Urban Areas, and Life Quality of Citizens—The Case of Warsaw

**DOI:** 10.3390/ijerph191710943

**Published:** 2022-09-02

**Authors:** Dagmara Stangierska, Iwona Kowalczuk, Ksenia Juszczak-Szelągowska, Katarzyna Widera, Weronika Ferenc

**Affiliations:** 1 Department of Pomology and Horticulture Economics, Institute of Horticulture Sciences, Warsaw University of Life Sciences—SGGW, Nowoursynowska 166, 02-787 Warsaw, Poland; 2 Department of Food Market and Consumer Research, Institute of Human Nutrition Sciences, Warsaw University of Life Sciences—SGGW, Nowoursynowska 159C, 02-776 Warsaw, Poland; 3 Department of Economics, Finance, Regional and International Research, Faculty of Economics and Management, Opole University of Technology, Prószkowska 76, 45-758 Opole, Poland

**Keywords:** green urban areas, urban environment, life quality, Poland

## Abstract

The increased migration of people from rural areas to cities has prompted researchers to take an interest in the problem of the quality of life (QOL) of the urban population in different contexts. The aim of the study was to determine the relationship between the level of satisfaction of Warsaw residents with urban infrastructure (SUI) and their QOL, the impact of the SUI on the perception of a neighborhood as an ideal place to live and the relationship between the amount of green areas and and the SUI of Warsaw residents and their QOL. The quantitative survey was conducted using the CAWI method on a sample of 381 adults. The WHOQOL-BREF questionnaire was used to measure QOL, the scale used in earlier surveys was used to assess SUI, areas of of Warsaw with different amounts of green space were distinguished using cluster analysis. The study showed a relationship between the SUI declared by residents and their QOL, mainly in the environmental domain. The discriminant analysis showed that satisfaction with greenery is one of the most important determinants of the subjective perception of a neighborhood as an ideal place to live. There was no direct effect of the amount of green areas in objective terms on the QOL of Warsawians, but a relationship was noted between the amount of green areas and SUI, with the highest level of satisfaction noted for the Green-balanced Cluster, characterized by the most favorable combination of quality and utility of urban area.

## 1. Introduction

Quality of life is an issue of interest to many sciences, including economics, sociology, medicine, psychology and pedagogy. This multidisciplinary approach has resulted in a variety of definitions of quality of life, depending on the paradigm adopted in a given science or by a given researcher [1]. In the 1960s and 1970s, an economic approach to assessing the quality of life was popular, according to which its level was determined by such objective criteria as income, the level of economic development, or the unemployment rate [2]. In opposition to this approach, other academics have begun to focus on the subjective experiences of individuals affecting their quality of life [3,4]. In attempting to reconcile the two concepts Smith (1973) [5] proposed that the term well-being should be used to refer to objective, measurable living conditions that affect entire populations, while the quality of life should be used to refer to the subjective feelings of individuals about the reality they experience. 

Currently, health, psychological, social and environmental criteria are most often taken into consideration when evaluating the quality of life, as reflected in the definition of quality of life proposed by the WHO, according to which quality of life is “a multifactorial construct containing domains of physical health, psychological well-being, social relationships, and the physical environment” [6]. Many conceptual and methodological analyses of QOL have been already published [7,8,9]. To summarize the approaches presented in literature, the area of health is constituted by variables such as energy and fatigue, pain and discomfort, sleep and rest, and physical activity. The psychological component is mainly composed of subjective factors, such as positive feelings, emotional well-being, spiritual well-being, challenges, prestige, personal development, humor, challenges, capabilities, understanding and solidarity, and sense of security [10]. Social determinants of quality of life most commonly include a position in the community or society, social participation, social relationships and friendship. Among environmental factors, issues related to satisfaction with areas of individual functioning such as culture and education, health care, tourism and recreation, the economy or the environment are considered [9,10,11]. 

The dynamic urbanization of European countries and the world in general has led researchers to take an interest in the quality of life of urban populations in both theoretical and cognitive terms. The quality of urban areas is increasingly recognised as an important determinant of the quality of life of its inhabitants [12]. 

Diagnoses of quality of urban life (QOUL) are made using both universal measurement scales (Quality of Life scale, Better Life Index, Happy Planet Index, World Happiness Report) and new research tools, taking into account the specificities of urban life and its conditions [13,14,15]. QOUL measurement scales in addition to universal quality of life indicators, taking into account aspects specific to the urban environment such as quality of the natural, social, built and economic environment, urban and suburban green spaces, public spaces and public buildings, culture, leisure, educational offer, health care, traffic and transportation. These aspects relate to the specific features of life in the city and constitute its broadly understood convenience [16]. 

Among these determinants of quality of life in urban spaces, urban green spaces deserve special attention. Taylor and Hochuli [17] stated two possible interpretations of green space: either an area with water or vegetation or an urban public open space with vegetation. Green space exposure can be defined as contact between green space and a human [18].The role of green spaces concerning QOUL has been demonstrated by some health science researchers [19,20] economics [21,22], urban planning and environmental science [23]. The research has shown that daily access to safe and good-quality green spaces encourages higher levels of physical activity among populations, as well as brings mental health benefits [24,25,26]. In addition to the health benefits, the researchers also point to other advantages resulting from the availability of green spaces [27,28], including environmental—maintaining biodiversity, regulation of humidity, mitigating climate change effects, regulation of temperature, rainwater retention, air purification, noise reduction, wind force reduction, creating shade [29,30,31], social—better interpersonal relationships, reduction in crime, reduction in domestic violence, increased comfort of living for local residents, development of local communities, a place for recreation and leisure [32,33] aesthetic—adding diversity to the landscape and breaking its monotony [34] and economic—increasing the value of properties; savings in heating and cooling costs [35]. 

Most large cities, including Warsaw, the capital of Poland, were formed as a result of the merger of the original cities with the surrounding suburban and rural areas, which led to the creation of metropolises, consisting of districts with different characteristics. The convergence and interaction of the functional, structural and social changes taking place in the urban agglomerations thus formed result in a spectrum of challenges to be met by the administrative authorities [36,37]. Research shows that often as a city grows, inequalities in access to urban amenities (including in the area of greenery) between inhabitants of different neighbourhoods begin to increase [38,39,40], which may be reflected, among other things, in their inhabitants’ different perceptions of quality of life.

The research carried out to date leads to the conclusion that when exploring the influence of the quality of infrastructure on the quality of life of the inhabitants of urban agglomerations, it is advisable not only to carry out analyses at a general level, but also to take into account the multifaceted nature of the issue. This multifaceted approach should concern, on the one hand, the specific impact of individual elements of infrastructure on the quality of life of inhabitants, and on the other hand, the impact of the heterogeneity of the city in terms of infrastructure on the quality of life of inhabitants in various areas of the city. It also seems important that the analyses include both the subjective feelings of inhabitants and the objective indicators specifying the issue of infrastructure-related quality of life [41,42,43,44,45,46].

In line with the above suggestions, the study attempted to determine: (1) the relationship between the level of satisfaction of Warsaw residents with urban infrastructure and their quality of life, (2) the influence of individual elements of infrastructure on the subjective perception of a given neighborhood as an ideal place to live, and (3) the relationship between the amount of green areas in each city area (defined using objective indicators) and the level of quality of life as well as satisfaction with various elements of urban infrastructure of residents in these areas.

Three main research hypotheses were formulated:

**Hypothesis** **1** **(H1).** *the level of quality of life of Warsaw residents depends on their satisfaction with selected elements of urban infrastructure,*

**Hypothesis** **2** **(H2).** *the amount of greenery in a city district is an important element of the urban infrastructure that influences the level of satisfaction of living in the district,*

**Hypothesis** **3** **(H3).** *the amount of green areas influences the level of satisfaction among Warsaw residents with urban infrastructure and their quality of life.*

## 2. Materials and Methods

### 2.1. Study Design and Participants

The paper is based on the results of a questionnaire research study conducted in the 2022 using CAWI method. The ethical aspects followed throughout the study ensured the continued safety of participants, as well as the integrity of the accumulated data. A brief description of the study and its objective, as well as the declaration of anonymity and confidentiality were given to the participants before taking the questionnaire. Respondents did not provide their names nor contact information (including the IP address) and could finish the survey at any stage. The answers were saved only when participants clicked the “submit” button after filling in the questionnaire.

The online survey was conducted in full observance of the national and international regulations compliant with the Declaration of Helsinki (2000). The personal information and data of the participants were anonymous, according to the General Data Protection Regulation of the European Parliament (GDPR 679/2016). The survey did not require approval by the ethics committee because of the anonymous nature of the online survey and the impossibility of tracking sensitive personal data.

Study participants were recruited among the people registered by personal contact and social media—they were adults who declared having lived in Warsaw for more than one year. Ultimately, the criteria assumed for selection were met by 381 people. 

### 2.2. Questionnaire

The research questionnaire consisted of three parts. Part one included questions on the quality of life-based on the WHO-developed WHOQOL-BREF questionnaire, with 26 questions relating to four domains of life:

- physical (domain 1–DOM1), including activities of daily living, dependence on medication and treatment, energy and fatigue, mobility, pain and discomfort, rest and sleep, and ability to work;
- psychological (domain 2–DOM2), involving physical appearance, negative and positive feelings, self-esteem, spirituality, religion and belief, thinking, learning, memory and concentration;
- social (domain 3–DOM3), taking into account personal relationships, social support, and sexual activity;
- environment (domain 4–DOM4), including elements such as financial resources, freedom, physical and mental safety, health and health care, the home environment, opportunities to acquire new information and skills, opportunities and participation in recreation and leisure activities, the physical environment (pollution, noise, traffic, climate), transport.

Responses to the WHOQOL-BREF scale were given on a scale of of 1 to 5.

The second part of the survey included 12 questions selected from a survey to measure the quality of urban life developed by the Türksevera et al. [15] and used by Pazhuhan et al. [14]. The questions included the respondents’ satisfaction with elements of urban infrastructure such as access to educational facilities, shopping centres and sports facilities, commuting, amount of green spaces, availability of children’s playgrounds and recreational facilities, night lighting, footpaths, cleanliness and aesthetics, noise levels, air quality and their opinion on the statement “my district is an ideal place to live”. All the questions were answered on a scale ranging from 1—totally disagree to 5—totally agree.

The third part of the questionnaire contained questions on respondents’ characteristics, accounting for gender, age, place of residence, education and income.

### 2.3. Characteristic of Respondents

In terms of gender, the sample consisted of 75.9% women and 24.1% men. The age structure of the studied group was: 18–25 years old—38.6%, 26–35 years old—21.8%; 36–55 years old—31.2%; 55 and older—8.4%. In terms of education, the largest group was people with higher education (66.7%), 31.8% of the respondents had completed vocational education and 1.1% had completed vocational or primary education. The analysis of the economic situation of the respondents showed that 15.2% of them had a monthly income of less than PLN 1500, 20.2% had an income in the range of PLN 1501–2500, 17.9% in the range of PLN 2501–4000, 29.2% of respondents declared an income within the range of PLN 4001–5500 and 15.6 earned more than PLN 5500 per person per month (Table 1).

### 2.4. Classification of Warsaw’s Districts according to the Amount of Green Areas

**Figure 1:**
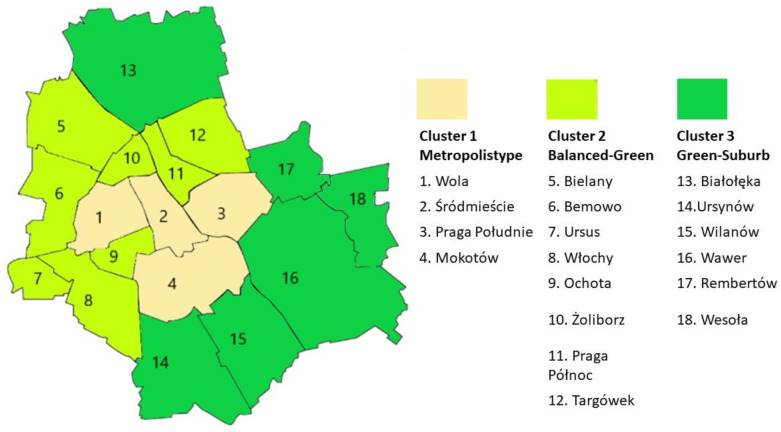
Cluster localization (the numbers on the map of the city of Warsaw indicate the districts whose names are given in the columns relating to the individual clusters).

In order to distinguish areas of Warsaw with different characteristics in terms of the amount of green areas, objective data on the city’s districts was taken into account, concerning: share of forests in the area of the district, the area of special natural value under legal protection, the share of green areas in the area of the district, the number of parks and green areas, balance of planting/loss of trees and shrubs, the share of agricultural and agricultural land in the area of the district. The districts were subdivided using Ward’s cluster analysis method. Application of hierarchical methods yielded a dendrogram, based on which three clusters of districts with the greatest similarity were distinguished. The first cluster (Metropolis type) consists of four city-center districts, characterized by a high proportion of green space and a large number of parks, a favourable ratio of planting to loss of trees and shrubs, and little forest and agricultural land. These districts comprised 27.8% of respondents. The second cluster (Balanced-Green) included nine districts located at some distance from the city centre, characterized by a low proportion of forests and agricultural land and moderate values for the other indicators considered in the analysis. The percentage of residents living in these districts was 27.1%. The third cluster (Green-Suburb) included six districts with the highest percentage of residents, 45.1%. These districts are characterized by a high proportion of forest and agricultural land, a large area of special natural value, a small number of parks and a low proportion of green space (Table 2, Figure 1).

### 2.5. Statistical Analysis

Statistical analysis of the empirical material included:

- determining the level of quality of life according to the methodology presented in the instructions for the WHOQOL-BREF scale [49] and converting the obtained results into a scale of 0 to 100 for each of the domains—based on the calculations made, it was found that the average quality of life score for the study population in the physical domain was 54.5, in the psychological domain 60.4, in the social domain 67.2, and in the environmental domain 67.6;
- determining differences in satisfaction ratings for the urban infrastructure elements included in the survey by respondents with high (50 and above) or low (below 50) quality of life scores in individual domains using Mann Whitney’s non-parametric U-test (in addition to the statistical values, mean values are also provided.);identifying the elements of urban infrastructure that determine the level of satisfaction with living in a given city district as a result of the construction of a discriminant model (Linear discriminant analysis -LDA), built for two groups of respondents: (1) those who disagree with the statement “my neighbourhood is the ideal place to live” (n^1^ = 109) and (2) those who agree with this statement (n_2_ = 288). In the adopted model, the Wilks’ λ- lambda discrimination coefficient was used to assess the discriminatory capacity of the variables under study, as well as the F-test and the χ^2^ test to verify the validity of the model obtained (α = 0.05). Elements of urban infrastructure such as educational facilities, shopping centres and sports facilities, commuting, amount of green spaces, availability of children’s playgrounds and recreational facilities, night lighting, footpaths, cleanliness and aesthetics, noise levels and air quality, measured on a rank scale, were used as discriminating variables. determining the relationship between the amount of green areas in individual districts of Warsaw (expressed as belonging to separate clusters) and the satisfaction of their residents with elements of urban infrastructure using the non-parametric Mann-Whitney U test (α = 0.05), in addition to the statistical values, mean values are also provided.

All calculations were made using the Statistica 14.1 program. 

## 3. Results

### 3.1. Satisfaction with Urban Infrastructure and Quality of Life

People with a lower level of quality of life (value 50 and below) in the physical domain were statistically significantly less satisfied with elements of urban infrastructure such as footpaths, access to educational facilities, commuting to work, the amount of greenery in the area and access to sports facilities (Table 3).

Respondents with lower levels of quality of life in the psychological domain were found to be statistically significantly less satisfied with elements of urban infrastructure such as commuting, footpaths, availability of shopping centers and educational facilities (Table 4). 

In the case of the social domain, people with lower levels of quality of life were statistically significantly less satisfied with all the elements of urban infrastructure included in the study except for noise level, air quality and neighbourhood cleanliness (Table 5).

Also concerning the environmental domain of quality of life, which is largely related to the state of infrastructure, there were statistically significant differences in their assessment by respondents with higher and lower levels of quality of life for almost all elements of infrastructure (except for air quality) (Table 6).

### 3.2. Urban Infrastructure Elements Determining the Perception of a Neighbourhood as an Ideal Place to Live

Discriminant analysis showed that among the eleven elements of urban infrastructure included in the study, satisfaction with footpaths, satisfaction with night lighting, dissatisfaction with air quality, satisfaction with the amount of green space, for which the values of the F statistic and the corresponding *p*-value are lower than the study’s statistical significance level of α = 0.05 (Table 7), were the strongest determinants of the perception of a neighbourhood as an ideal place to live.

The Wilks’ λ-lambda coefficient is in the interval [0; 1]. The smaller its value, the greater the ability of the variable to discriminate the set of respondents adopted for the analysis. F-statistic values for the variables: Satisfaction with Footpaths, Satisfaction with night lighting, Dissatisfaction with air quality, Satisfaction with the amount of green space (respondents’ answers to the statements) listed in Table 8 and the corresponding *p*-values are smaller than the statistical significance level α = 0.05 assumed in the study. This means that all variables listed in Table 8 are characterised by a statistically significant discriminatory power of the set of respondents studied. In order to assess the discriminating power of the estimated function, a canonical analysis was then performed, the results of which, presented in Table 8, testify to the high discriminating power of the discriminating model described by the discriminating variables listed in Table 7. The 80.32% classification validity score obtained on the basis of the model in Table 7 testifies to a high level of fit.

The value of Wilks’ λ- lambda coefficient in Table 8 equal to 0.6747 informs about high separating power of the discriminating model described by the variables listed in Table 7. The value of *χ*^2^ statistic and the corresponding *p*-value is lower than the significance level assumed in the study, which means that all variables from Table 7 obtained the status of discriminating variables in the studied model. Finally, the so-called standardised coefficients of the canonical discriminant function were calculated, the values of which supplement the information on the discriminant properties, indicated in Table 8 of the variables. The higher their absolute value, the better a given variable is than the others as a “discriminator” of respondents into groups of respondents considering their neighbourhood as an ideal place to live and not considering their neighbourhood as an ideal place to live. Table 9 shows the discriminating variables in order of their discriminating power. The results obtained indicate that, of the urban infrastructure elements analysed, the greatest influence on the perception of the neighbourhood as an ideal place to live on the change in preference of the neighbourhood of residence is the level of satisfaction with pedestrian paths and the level of satisfaction with the second strongest determinant is satisfaction with the amount of green space.

### 3.3. Amount of Green Space Versus Satisfaction with Urban Infrastructure

Analysing the relationship between the objectively measuredamount of green areas (expressed in the form of clusters with different characteristics in this regard) and the quality of life of Warsaw residents representing individual clusters, no statistically significant differences were found in the case of any of the domains. As far as respondents’ satisfaction with the urban infrastructure was concerned, statistically significant differences between separate clusters concerned such issues, as availability of children’s playgrounds and recreational facilities, amount of green space night lighting, noise levels, cleanliness and aesthetics, air quality and access to shopping centers. Almost all of the listed elements were rated the highest by representatives of the Balanced-green cluster. The exceptions were cleanliness and aesthetics, with which the representatives of the Green-Suburb and Balanced-Green clusters were equally satisfied, and access to shopping centres, with which the representatives of the Metropolis type and Balanced Green clusters were equally satisfied (Table 10).

## 4. Discussion

One of the three main objectives of the study was to determine the relationship between the level of satisfaction of Warsaw residents with urban infrastructure and their quality of life. The results obtained indicate that satisfaction with individual elements of urban infrastructure is most strongly reflected in the environmental aspect of quality of life, to a lower extent in the social aspect, and has relatively little impact on the physical and mental dimensions of wellbeing. Grum and Grum [50] also found a significant relationship between the analysed indicators of urban infrastructure (classified as social infrastructure in their analysis) and quality of life. Similar conclusions were also found by other researchers, who pointed out the importance of such elements of urban infrastructure for wellbeing, as the organisation of urban transport [51], necessary services and enough public space avaliability [52] or the availability of kindergartens and crèches, medical care and sports facilities [53]. In the research carried out, the strongest strength of relationship with the environmental domain of quality of life was found for satisfaction with footpaths. The importance of the availability and quality of footpaths has also been raised in other studies [54,55,56] which show that in an era of increasing health and environmental consciousness, pedestrian-friendly transport options are becoming increasingly important to city inhabitants.

An important factor affecting not only the environmental, but also other domains of quality of life is commuting. A study by Chatterjee et al. [57] has shown that their proper organisation from the point of view of broader commuting solutions can be an important determinant of the wellbeing of city inhabitants, mainly by reducing the level of stress resulting from time pressure and the unpredictability of problems that can occur during the daily commute [58].

Also, the availability of children’s playgrounds, recreational and sport facilities positively correlates with an assessment of the environmental domain of quality of life. Studies have shown that spending time actively by children promotes their physical, social and emotional development [59], and the appropriate amount and organization of urban recreational areas play a significant role in increasing recreational wellbeing, which is positively related to individuals’ physical well-being associated with happiness and life satisfaction [60]. Many studies indicate a positive correlation between physical activity and subjective well-being [61,62]. Sport may also protect against symptoms of mental disorders that are increasingly prevalent among adolescents [63]. In addition, Downward and Rasciute [64] have shown that, in addition to a positive effect upon the subjective well-being of individuals, participation in sport has a beneficial effect on social relationships.

Another element of urban infrastructure positively correlating with environmental aspects of quality of life was the availability of educational facilities. According to Opris and Necsulescu [65] educational amenities are one of the main features of urban structure that need to be addressed when planning development at urban or zoning level. The research shows that the availability of educational facilities is of particular importance to parents of school-age children and young adult students. Easy access to educational facilities is an important factor in choosing where to live, which translates into housing demand, and consequently prices [66].

The aesthetics and cleanliness of urban areas was also an important aspect of urban infrastructure determining the level of quality of life in the environmental domain. The importance of the aesthetics of urban solutions is also highlighted by Ellard [67] in that it can have an impact on improving the general atmosphere and removing barriers to social cohesion. According to other researchers [68,69] access to well-maintained public spaces can contribute to the physical and mental well-being of city inhabitants, so urban aesthetics deserve special attention [70].

An important element influencing the aesthetics of urban spaces are green areas [34]. Although the strength of the correlation between satisfaction with the amount of green areas and quality of life found in the study was low, statistically significant differences were found in the assessment of satisfaction with the amount of green areas for people with low and high quality of life in the physical, social and environmental domains This multi-directional impact of urban greenery is confirmed in other research, which highlight the positive effects of greenery on health [24]; the environment [29,30,31], social relations [32,33] and the economy [35].

Given the relationship found between satisfaction with urban infrastructure and quality of life, it can be assumed that the first of the research hypotheses was proven as a result of the study.

The second objective of the study was to determine which elements of the urban infrastructure most strongly influence the perception of a neighborhood as an ideal place to live. The results indicate that the factors most strongly determining this perception of a place to live are the level of satisfaction with footpaths and the level of satisfaction with the amount of green space, confirming the assumption made in hypothesis two. Both of these infrastructure elements were among the factors influencing the assessment of quality of life, which to a large extent explains their positive influence on the perception of the place of residence, and this is consistent with other research findings [71,72,73,74]. The issue of greenery was also considered important in other studies aimed at identifying the attributes of the best places to live, although more importance was attributed to factors not included in the survey conducted, such as a sense of community, neighborhood and a sense of safety and security, the cost of housing and satisfaction with the location of housing [45,75,76,77,78,79,80].

The third area of analysis was the impact of the objectivly measured amount of green space on quality of life and satisfaction with urban infrastructure. The analyses carried out did not show a direct relationship between the amount of greenery (expressed in the form of Clusters with different characteristics in this respect) and the quality of life of residents, which was also noted in other studies [81]. On the other hand, a relationship was found between the amount of green areas and respondents’ satisfaction with the elements of urban infrastructure included in the study, which constitutes a partial positive verification of hypothesis 2.

The highest level of satisfaction for almost all assessed elements of urban infrastructure was declared by the Balanced-green cluster. This cluster is made up of nine neighbourhoods located at some distance from the city centre. In terms of green areas, this cluster is characterised by a small share of forests and agricultural land, a small area of special nature value under legal protection, a small number of parks and green spaces, a poor balance of planting/loss of trees and shrubs, but a relatively high share of green areas in the area of the district. The districts forming the cluster are well connected to the rest of the city, offer a full range of public services and good commercial infrastructure. The parameters characterising environmental quality (noise, pollution) in the area are moderately favourable. The Balanced—green cluster can therefore be considered to have a good mix of different elements of urban infrastructure and the best combination of environmental quality and utulity in relation to the other clusters. The fact that it was the representatives of this cluster who appeared to be the most satisfied with the infrastructure and, albeit on average, the happiest confirms the view commonly presented in the scientific literature [82,83,84,85,86] that the condition for a high level of urban life quality is the balance between all the elements that make up urban space.

## 5. Strengths, Limitations and Future Research

The obtained results can be important both form the cognitive and applied point of view. To the best of the authors’ knowledge, the correlation between satisfaction with urban infrastructure and quality of life in Poland’s capital has not yet been studied, and the method used to classify the districts that make up the urban area in terms of the amount of green space has not yet been applied. The results of the study may provide a basis for modifying the perception of the importance of the analyzed aspects and urban space by the institutions responsible for its shaping.

A certain limitation of the study is the fact that it concerns the residents of Warsaw, which calls for confirming the observed correlations with studies in other cities and countries. It would also be advisable to make future research more detailed, on the one hand by taking into account the demographic, social and economic characteristics of the respondents, and on the other by including more elements in the set of variables describing infrastructure.

In future research, it would be worthwhile not only to focus on classifying districts/cities according to the amount of greenery, but to try to reflect the quality of this greenery in terms of its usefulness to residents (functions and frequency of use).

## 6. Conclusions

The study showed a correlation between satisfaction with such elements of urban infrastructure as pedestrian paths, commuting, availability of children’s playgrounds and recreational facilities, access to educational facilities, accessibility of sports facilities, cleanliness and aesthetics and residents’ quality of life, mainly in the environmental domain. The discriminant analysis showed that satisfaction with greenery is one of the most important determinants of the perception of a neighbourhood as an ideal place to live. However, there was no effect of the amount of green areas (expressed in the form of Clusters with different characteristics in this regard) on the quality of life of Warsaw’s residents, but a relationship was noted between the amount of green areas and satisfaction with individual elements of urban infrastructure, with the highest level of satisfaction noted for the Green-balanced Cluster, characterized by the most favorable combination of quality and utulity of urban area.

The results show that subjective satisfaction with the amount of greenery influences both the assessment of quality of life and the perception of the neighbourhood as an ideal place to live. However, from an objective point of view, there was no correlation between the amount of greenery and quality of life or satisfaction with urban infrastructure, suggesting that in order to improve the wider quality of life for city dwellers, it is necessary not only to objectively increase the amount of green space in urban areas, but above all to make them more attractive and maximise their accessibility for residents.

## Figures and Tables

**Table 1:** Sample characteristics (%).

**Gender**				
Female		Male		
75.85		24.15		
**Age**				
18–25	26–35	36–55	Over 55	
38.58	21.78	31.23	8.41	
**Education**				
Primary	Vocational	Secondary	Higher	
0.52	1.05	31.71	66.62	
Per Capita Income PLN (EUR) *				
Under 1500 (315)	1501–2500 (315.1–525.2)	2501–4000 (525.3–840.3)	4001–5500 (840.4–1155.5)	Over 5500 (1155.5)
15.21	20.21	17.85	29.18	17.55

* As of 21.07.2022 [47].

**Table 2:** Clusters characteristic.

Specyfication *	Factor Weighting from Cluster Analysis	Cluster 1 Metropolis-Type n = 106	Cluster 2 Balanced–Green n = 103	Cluster 3 Green–Suburb n = 172
Share of forests in the area of the district (in % of area)	100	0.35	1.76	14.08
Area of special nature value under legal protection (in ha)	100	205.03	244.53	1574.97
Share of green areas in the area of the district (in % of area)	99	16.33	14.28	0.35
Number of parks and green areas	98	47.00	16.13	8.00
Balance of planting/ loss of trees and shrubs	98	17,993.00	2551.63	3361.67
Share of agricultural and agricultural land in the area of the district (in % of area)	74	1.08	1.63	13.82

* As of [48].

**Table 3:** Level of satisfaction with selected elements of urban infrastructure concerning the level of quality of life in the physical domain (DOM1) (Mann-Whitney U test).

Specification	Lower Level of QOL DOM1 (Average Score)	Higher Level of QOL DOM1 (Average Score)	Z (U Mann-Whitney)
Satisfaction with access to educational facilities.	3.41	3.72	−2.92 *
Satisfaction with access to shopping center’s.	3.88	4.08	−1.83
Satisfaction with commuting	3.58	4.03	−3.91 *
Satisfaction with the amount of green space	3.64	4.03	−2.67 *
Satisfaction with the availability of children’s play grounds and recreational facilities	3.60	3.82	−1.78
Satisfaction with the accessibility of sports facilities	3.42	3.68	−2.48 *
Satisfaction with night lighting	3.63	3.77	−1.24
Satisfaction with Footpaths	3.67	3.91	−2.24 *
Satisfaction with the cleanliness and aesthetics	3.42	3.64	−1.72
Satisfaction with noise levels	3.29	3.45	−1.05
Dissatisfaction with air quality	3.59	3.53	0.67

* *p*-value < 0.05.

**Table 4:** Level of satisfaction with selected elements of urban infrastructure concerning the level of quality of life in the psychological domain (DOM2) (Mann-Whitney U test).

Specification	Lower Level of QOL DOM2 (Average Score)	Higher Level of QOL DOM2 (Average Score)	Z (U Mann-Whitney)
Satisfaction with access to educational facilities	3.34	3.68	−2.89 *
Satisfaction with access to shopping center’s	3.71	4.09	−3.29 *
Satisfaction with commuting	3.39	4.00	−4.60 *
Satisfaction with the amount of green space	3.76	3.91	−1.37
Satisfaction with the availability of children’s play grounds and recreational facilities	3.58	3.78	−1.80
Satisfaction with the accessibility of sports facilities	3.40	3.64	−1.90
Satisfaction with night lighting	3.60	3.75	−1.68
Satisfaction with footpaths	3.58	3.90	−2.66 *
Satisfaction with the cleanliness and aesthetics	3.38	3.61	−1.86
Satisfaction with noise levels	3.26	3.43	−1.16
Dissatisfaction with air quality	3.47	3.57	−0.90

* *p*-value < 0.05.

**Table 5:** Level of satisfaction with selected elements of urban infrastructure concerning the level of quality of life in the social domain (DOM3) (Mann-Whitney U test).

Specification	Lower Level of QOL DOM3 (Average Score)	Higher Level of QOL DOM3 (Average Score)	Z (U Mann-Whitney)
Satisfaction with access to educational facilities.	3.22	3.69	−3.57 *
Satisfaction with access to shopping center’s	3.66	4.09	−3.12 *
Satisfaction with commuting	3.25	4.01	−4.70 *
Satisfaction with the amount of green space	3.62	3.94	−2.32 *
Satisfaction with the availability of children’s playgrounds and recreational facilities	3.47	3.80	−2.54 *
Satisfaction with the accessibility of sports facilities	3.34	3.64	−2.46 *
Satisfaction with night lighting	3.40	3.80	−2.77 *
Satisfaction with Footpaths	3.53	3.89	−2.64 *
Satisfaction with the cleanliness and aesthetics	3.38	3.60	−1.76
Satisfaction with noise levels	3.26	3.42	−1.05
Dissatisfaction with air quality	3.43	3.58	−0.82

* *p*-value < 0.05.

**Table 6:** Comparison of satisfaction with selected elements of urban infrastructure by level of quality of life in the environmental domain (DOM4) (Mann-Whitney U test).

Specification	Lower Level of QOL DOM4 (Average Score)	Higher Level of QOL DOM4 (Average Score)	Z (U Mann-Whitney)
Satisfaction with access to educational facilities.	3.17	3.66	−2.91 *
Satisfaction with access to shopping centres.	3.47	4.07	−3.36 *
Satisfaction with commuting	3.30	3.93	−3.41 *
Satisfaction with the amount of green space	3.49	3.93	−2.49 *
Satisfaction with the availability of children’s playgrounds and recreational facilities	3.19	3.81	−3.20 *
Satisfaction with the accessibility of sports facilities	3.09	3.65	−2.98 *
Satisfaction with night lighting	3.43	3.76	−1.92
Satisfaction with footpaths	3.02	3.93	−4.61 *
Satisfaction with the cleanliness and aesthetics	3.02	3.63	−3.39 *
Satisfaction with noise levels	2.98	3.45	−2.26 *
Dissatisfaction with air quality	3.74	3.52	1.49

* *p*-value < 0.05.

**Table 7:** Results of the discriminant analysis.

N = 381	Lambda Wilksa	Particle Wilksa	F Moved. (1.34)	*p*-Value	Tolerance	1-Tolerance (R-Kwad)
Satisfaction with footpaths	0.7716	0.8744	54.0252	0.0000	0.8522	0.1478
Satisfaction with night lighting	0.6856	0.9840	6.1107	0.0139	0.8611	0.1389
Dissatisfaction with air quality	0.6888	0.9796	7.8456	0.0054	0.9563	0.0437
Satisfaction with the amount of green space	0.6979	0.9667	12.9436	0.0004	0.8667	0.1333

**Table 8:** Results of the canonical analysis.

Moved	Own Value	Canonical R	Wilksa Lambda	*χ* ^2^	df	*p*-Value
0	0.4822	0.5704	0.6747	148.3590	4	0.0000

**Table 9:** Standardised coefficients of the canonical discrimination function for discriminating variables.

**Variable (Statement)**	**Satisfaction with Footpaths**	**Satisfaction with the Amount of Green Space**	**Dissatisfaction with Air Quality**	**Satisfaction with Night Lighting**
Value of the standardised coefficient	0.6732	0.34368	−0.2563	0.2389

**Table 10:** Level of satisfaction with urban infrastructure in clusters with different characteristics in terms of amount of green space (Mann-Whitney U test for pairs of clusters).

Specification	Cluster 1 Metropolistype (Average Score)	Cluster 2 Balanced–Green (Average Score)	Cluster 3 Green–Suburb (Average Score)	Z (U Mann-Whitney) Cluster 1 vs. Cluster 2	Z (U Mann-Whitney) Cluster 1 vs. Cluster 3	Z (U Mann-Whitney) Cluster 2 vs. Cluster 3
Satisfaction with access to educational facilities.	3.56	3.67	3.58	−1.09	−0.37	0.85
Satisfaction with access to shopping center’s	4.18	4.15	3.80	0.08	2.75 *	2.62 *
Satisfaction with commuting	4.06	3.82	3.75	1.28	1.82	0.37
Satisfaction with the amount of green space	3.74	4.20	3.77	−2.92 *	−0.18	3.01 *
Satisfaction with availability of children’s playgrounds and recreational facilities	3.68	4.04	3.58	−2.54 *	0.70	3.37 *
Satisfaction with accessibility of sports facilities	3.51	3.76	3.52	−1.69	−0.19	1.64
Satisfaction with night lighting	3.68	3.88	3.64	−1.94	−0.05	2.11 *
Satisfaction with footpaths	3.83	3.89	3.77	−0.32	0.45	0.83
Satisfaction with the cleanliness and aesthetics	3.30	3.64	3.66	−2.17 *	−2.35	0.30
Satisfaction with noise levels	3.36	3.59	3.28	−1.16	0.77	2.17 *
Dissatisfaction with air quality	3.84	3.27	3.53	3.79 *	2.35 *	−1.87

* *p*-value < 0.05.

## Data Availability

The authors confirm that the datasets analyzed during the study are available from the corresponding author upon reasonable reques.
